# Are the Properties of Bone Marrow-Derived Mesenchymal Stem Cells Influenced by Overweight and Obesity?

**DOI:** 10.3390/ijms24054831

**Published:** 2023-03-02

**Authors:** Qiang Zong, Katrin Bundkirchen, Claudia Neunaber, Sandra Noack

**Affiliations:** Hannover Medical School, Department of Trauma Surgery, Carl-Neuberg-Str. 1, 30625 Hannover, Germany

**Keywords:** overweight, obesity, BMSCs, proliferation, clonogenicity, surface antigen, senescence, apoptosis, differentiation

## Abstract

Bone marrow-derived mesenchymal stem cells (BMSCs) are promising candidates for cell-based therapies. Growing evidence has indicated that overweight/obesity can change the bone marrow microenvironment, which affects some properties of BMSCs. As the overweight/obese population rapidly increases, they will inevitably become a potential source of BMSCs for clinical application, especially when receiving autologous BMSC transplantation. Given this situation, the quality control of these cells has become particularly important. Therefore, it is urgent to characterize BMSCs isolated from overweight/obese bone marrow environments. In this review, we summarize the evidence of the effects of overweight/obesity on the biological properties of BMSCs derived from humans and animals, including proliferation, clonogenicity, surface antigen expression, senescence, apoptosis, and trilineage differentiation, as well as the underlying mechanisms. Overall, the conclusions of existing studies are not consistent. Most studies demonstrate that overweight/obesity can influence one or more characteristics of BMSCs, while the involved mechanisms are still unclear. Moreover, insufficient evidence proves that weight loss or other interventions can rescue these qualities to baseline status. Thus, further research should address these issues and prioritize developing methods to improve functions of overweight- or obesity-derived BMSCs.

## 1. Introduction

Overweight and obesity are conditions in which a person’s total body weight exceeds what is considered healthy [1]. Body mass index (BMI) is widely used as an indicator to evaluate weight status. Based on BMI, adults are usually categorized as underweight (<18.5 kg/m^2^), normal (18.5–24.9 kg/m^2^), overweight (25–29.9 kg/m^2^), and obese (≥30 kg/m^2^) [2,3]. BMI percentile can be used to evaluate the overweight/obese status of children and adolescents [4]. Over the past few decades, the global prevalence of overweight/obesity has reached a pandemic level [5,6]. The World Health Organization (WHO) reported that worldwide obesity has nearly tripled since 1975 [7,8]. As troubling as these numbers are, future trends are even more alarming. Up to 45% of the global population is predicted to be overweight and 16% obese by 2050 [9]. Overweight and obesity are recognized as major global public concerns resulting in significant medical and economic burdens on nations and individuals. They increase the risk of numerous health problems including diabetes, hypertension, stroke, and heart failure [10,11,12,13,14]. It was reported that the general mortality rate increases by 30% for every increase of 5 kg/m^2^ in BMI among individuals with a BMI greater than 25 kg/m^2^ [15].

Bone marrow-derived mesenchymal stem cells (BMSCs) are one of the most attractive seed cells in tissue engineering and regenerative medicine [16,17,18]. They are capable of self-renewal and proliferate as plastic-adherent cells in vitro with fibroblast-like morphology [19]. Additionally, these cells can form colonies in vitro while retaining the potential to differentiate into multiple mesenchymal lineages, such as osteoblast, adipocyte, and chondrocyte [20]. Current studies have revealed that BMSCs are able to promote tissue repair, modulate inflammation and immunity, and support hematopoiesis [21,22,23,24,25,26,27]. Benefiting from their prominent properties and functions, a great number of clinical trials based on BMSCs are in progress around the world.

With the unprecedented increase in the incidence of overweight and obesity, the overweight and obese population will inevitably become potential BMSC donors. This could be a problem as many researchers believe that the increase in unhealthy body weight can lead to abnormal properties and functions of BMSCs. Therefore, it is important to evaluate the quality of overweight/obesity-derived BMSCs to ensure their safety and efficacy. However, current findings from animal and human studies on the effects of overweight/obesity on BMSCs are inconsistent. Several studies demonstrate that excess body weight leads to undesirable proliferation, differentiation, and self-renewal of BMSCs [28,29], yet some other studies fail to provide support for these conclusions [30,31,32]. In short, the potential impact of overweight and obesity on BMSCs is still unclear and controversial. Therefore, this review summarizes the influence of overweight or obesity on human or animal BMSC biology, including proliferation, clonogenicity, surface antigen expression, senescence, apoptosis, trilineage differentiation, and the underlying mechanisms (included studies are shown in Table 1 and Table 2). It will provide researchers with more insight into the changes in the biological properties of BMSCs under overweight/obese conditions and give valuable references for subsequent clinical translation.

## 2. Effect on Proliferation and Colony-Forming Ability of BMSCs

The number of mesenchymal stem cells in the bone marrow is very low, ranging from 0.001–0.01% of nucleated cells [53]. The in vitro proliferative capability of BMSCs is very important because a high number of cells are required for clinical use. Clonogenicity is a common method to assess the self-renewal of mesenchymal stem cells (MSCs) [54]. Nowadays, colony formation efficiency is often employed to evaluate the quality of MSC preparations for preclinical studies and clinical trials [55,56]. In animal research, high-fat diet (HFD)-induced obesity best mimics the physiological functions of the obese human body [33,57]. These animals have typical features of human obesity such as central adiposity, hyperinsulinemia, and insulin resistance [58,59]. Benova et al. [48] demonstrated that the short-term proliferation ability between BMSCs from obese and lean mice is not significantly different. Wu et al. [30] found a similar result in BMSCs cultured through five passages under hypoxic conditions. However, da Silva et al. [36] observed a higher proliferative activity in BMSCs from obese mice than BMSCs from lean mice when the cells were unstimulated at baseline. In contrast, stimulation with fibroblast growth factor-2 (FGF-2, 3 ng/mL) for 48 h significantly enhances the proliferation of lean BMSCs but does not lead to a further increase in the proliferation capacity of obese BMSCs. This seems to indicate that the proliferative responses of obese BMSCs have reached a plateau, which is reasonably speculated to be associated with the long-term stimulation of the high concentration of FGF-2 caused by obesity [60,61,62]. A study has shown that the proliferative ability of human BMSCs (hBMSCs) stimulated with FGF-2 (3 ng/mL) for a long time exhibits a biphasic response—rising first and then falling [63]. Zhu et al. [64] found that a long-term high-fat diet downregulated the level of FGF-2 in mouse BMSCs. FGF-2 has been reported to promote the proliferation of BMSCs [63,65], while low-expressed FGF-2 induced by a 6-month high-fat diet did not harm the abundance and proliferative rate of BMSCs in the study of Zhu et al. [64], suggesting that BMSCs proliferation is subject to multifaceted regulatory pathways. However, Alessio et al. [42] reported that the proliferation potential of BMSCs from obese mice is impaired. The cell cycle analysis of this study also shows that obesity decreases the proportion of BMSCs in the S phase. The S phase is the period of DNA replication during cell proliferation, which occurs between the G1 and G2 phases [66]. The reduced ratio of cells in the S phase usually reflects suppressed cell proliferative capacity. Consistent with this, the levels of cell cycle inhibitors p16 and p53 proteins in obese BMSCs markedly increased compared with those in the lean BMSCs [42]. These results were bolstered by another study, in which Bi et al. [44] presented that BMSCs from female Sprague–Dawley (SD) rats with significantly increased body weight induced by 4- or 6-month HFD have a longer doubling time along with G1/G0 arrest than BMSCs from female SD rats fed a normal diet. Likewise, Tencerova et al. [38] observed inhibited proliferation capacity in obese mouse BMSCs. Furthermore, they reported that obese BMSCs exhibit attenuated colony-forming potential compared with lean BMSCs [38]. In the study of Li et al. [41], the inhibitory effect of obesity on the clonogenicity of mouse BMSCs was also noted, which can be reversed by the knockout of the interleukin 6 (*IL-6*) gene. However, there are still animal experiments manifesting that obesity does not interfere with the colony-forming ability of BMSCs [32,35,42,48].

To our knowledge, studies on the effects of high BMI (overweight/obesity) on the proliferation and colony-forming ability of hBMSCs are limited. Real-time cell analysis employed by Ulum et al. [28] shows that the proliferation rate of hBMSCs from donors with high BMI (BMI > 30 kg/m^2^) is slower than that of hBMSCs from donors with normal BMI (BMI < 25 kg/m^2^). In a study focusing on obesity increasing bone fragility, Tencerova et al. [29] compared obese and overweight hBMSCs with lean hBMSCs. They found that both obesity and overweight attenuate the short-term proliferation potential of hBMSCs. However, Di Bernardo et al. [52] obtained a different result in a pilot study when they determined the role of overweight on the proliferation from the aspect of a circulating signaling molecule. hBMSCs from three male donors (aged 10, 12, 13 years) were co-cultured with serum from overweight (BMI > 25 kg/m^2^) and healthy (BMI < 25 kg/m^2^) adults, which displayed a comparable proliferation rate [52]. This finding is inconsistent with the results of the study by Tencerova et al. [29], which might be attributed to the difference in sample size, culture conditions, or cell sources. As for the colony-forming ability, Tencerova et al. [29] found that the colony number of obese hBMSCs significantly decreases compared with that of lean hBMSCs, which is in line with the diminished stemness of obese hBMSCs suggested by RNA sequencing. Nevertheless, overweight was not found to be detrimental to the clonogenic potential of hBMSCs [29]. This discrepancy might reflect different bone marrow microenvironments created by overweight and obesity. In another study, McCann et al. [51] investigated hBMSCs from adult patients undergoing hip replacement surgery. Interestingly, they found that total colony area and mean colony area significantly rise with increasing BMI in all human participants. After grouping by gender, however, total colony amount and mean and total colony area are positively correlated with BMI in males but not females. Moreover, linear regression analysis reveals that BMI can strongly predict colony area and number in males. This indicates that the effect of BMI on colony-forming efficiency may be gender-specific. Therefore, it must be recognized that the adult stem cell population can be simultaneously influenced by various aspects such as gender, BMI, and age when considering hBMSC-based therapies. If necessary, any point needs to be analyzed independently.

## 3. Effect on Surface Antigen Expression of BMSCs

The expression of surface antigens on BMSCs reflects cellular biological functions and basic characteristics such as cell differentiation, proliferation, clonogenicity, and aging [67]. Therefore, elucidating the alteration of the surface antigen profile on BMSCs under obese/overweight conditions helps understand the underlying mechanisms of changes in cellular properties. Several studies have shown that BMSCs from animals or humans with excess weight normally express common cell surface antigens [29,36,40,42]. However, Wu et al. [30] found that obese mice have a reduced percentage of BMSCs expressing CD105 (CD: Cluster of Differentiation) and the percentage expressing platelet-derived growth factor receptor α (PDGFRα, CD140a) increased compared with lean mice. These alterations may contribute to impaired chondrogenesis of obese BMSCs [30]. In contrast, Tencerova et al. [38] demonstrated that the percentage of CD73+ and Sca1 (Stem cells antigen-1)/CD140a+ cells is decreased in obese mouse BMSC cultures, suggesting that the percentage of progenitor cell populations might have changed [68]. Picke et al. [32] found that BMSCs from obese mice express less CD90 than those from lean mice, which might be associated with increased tumor necrosis factor-α induced by obesity. Compared with wild-type BMSCs, CD90-deficient BMSCs display enhanced adipogenic but inhibited osteogenic differentiation potential [32]. The attenuated osteogenesis has been linked to the disturbed Wnt signaling pathway led by CD90 loss [32]. Moreover, CD90-depleted BMSCs have increased proliferation and cell growth but a trend toward diminished apoptosis, which illustrates that CD90 regulates the properties of BMSCs through multiple directions.

In humans, Ulum et al. [28] compared hBMSCs from high-BMI (BMI > 30 kg/m^2^) and normal-BMI (BMI < 25 kg/m^2^) donors. They also found reduced CD90 expression on hBMSCs in the high BMI group. Yet these hBMSCs exhibit suppressed proliferation ability, which might be due to more molecular changes involved because the expressions of CD73, CD105, CD29, CD44, and CD166 are downregulated but CD31 is upregulated on the surface of hBMSCs from donors with high BMI [28]. By contrast, Tencerova et al. [29] found no significant differences in the expressions of CD44, CD90, and CD105 between obese or overweight patient- and lean patient-derived hBMSCs. On the other hand, obese patients have an increased number of cells expressing leptin receptor (LEPR), insulin receptor (IR), and C-X-C chemokine receptor (CXCR4) compared with lean patients. These antigens are related to cell proliferation, clonogenicity, lineage commitment, and aging [29,69,70,71]. However, no significant change was detected in the proportion of LEPR+, IR+, and CXCR4+ cells between overweight and lean participants. Therefore, differential effects of overweight and obesity on BMSCs are also reflected in the expression of surface antigens, which needs to be further studied.

## 4. Effect on Senescence of BMSCs

The influence of obesity and overweight on the aging phenotype of BMSCs is a critical issue that has not been fully clarified. Cellular senescence, a state of irreversible growth arrest, can limit the regeneration potential of MSCs [72,73]. Clinically applying senescent MSCs may not yield the expected results and even lead to undesirable consequences [74,75,76,77,78]. An animal experiment reports that HFD-induced obesity does not result in the aging phenotype of mouse BMSCs, as the number of senescence-associated β-galactosidase-positive (β-gal+) cells and levels of senescence-related markers p16 and p21 mRNA in obese BMSC cultures are similar with those in lean BMSC cultures [38]. However, most experiments still suggest that obesity accelerates the senescence of BMSCs. Alessio et al. [42] demonstrated that obese mice have a higher percentage of senescent BMSCs than normal mice. As the classic hallmarks of cellular aging [79,80], a high level of intracellular reactive oxygen species (ROS) was detected in obese BMSC cultures [42]. Moreover, obesity also upregulates the protein expression of canonical senescence makers p53 and p16 (Rb and p21 proteins unchanged) in obese BMSCs [42]. In a recent study, Li et al. [41] did not find a difference in the level of p16 mRNA between obese and control wild-type (WT) mouse BMSCs, while p53 and p21 proteins significantly increased in obese cells. Meanwhile, obesity elevates the concentration of IL-6 in the WT mouse-derived BMSC supernatant [41]. IL-6 is one of the most prominent cytokines of the senescence-associated secretory phenotype [81]. The increase in IL-6 suggests obesity-induced BMSC senescence, which might be associated with the IL-6/STAT3 pathway [41]. In another study on female SD rats, Bi et al. [44] found that higher body weight contributes to BMSC aging accompanied by high ROS levels and increases the serum level of C-X-C motif chemokine ligand 2 (CXCL2). These BMSCs exhibit a typical sign of aging that the cells remain halted at the G1 phase [82]. An in vitro experiment proves that CXCL2 can lead to BMSC senescence by enhancing oxidative stress [44]. By means of RNA sequencing, Ali et al. [47] found enrichment of MAPK and JAK–STAT target genes in BMSCs of obese ovariectomized (OVX) female mice, which are involved in cellular senescence.

In humans, Di Bernardo et al. [52] investigated the potential effects of circulating factors from overweight subjects (BMI > 25 kg/m^2^) on the senescence process of hBMSCs. The β-galactosidase assay shows that hBMSCs treated with overweight serum have a similar senescence rate with cells treated with healthy (BMI < 25 kg/m^2^) serum. However, Ulum et al. [28] demonstrated that the senescence level in hBMSCs is significantly higher in high BMI (BMI > 30 kg/m^2^) samples than in normal BMI (BMI < 25 kg/m^2^) controls. In terms of morphology, they found that hBMSCs from donors with high BMI are more cubiform and larger compared with cells from donors with normal BMI [28]. Generally, senescent MSCs often exhibit enlarged or flattened morphology [82]. However, this morphological alteration under high BMI conditions has not been verified in laboratory animal models. A few animal experiments report that the overall morphology of BMSCs derived from HFD-induced obese mice and lean mice is similar, exhibiting a typical spindle-shaped phenotype [30,36]. Tencerova et al. [29] also presented that obesity can promote hBMSCs’ aging confirmed by increased senescent β-gal+ cells, higher β-gal activity, an elevated level of ROS production, and upregulated expression of senescence-associated genes and oxidative stress markers in obese cell cultures [83,84]. The senescence phenotype is characterized by enriched IR+ and LEPR+ hBMSCs and is associated with enhanced insulin signaling. This phenotype can be reversed by blocking insulin signaling in obese hBMSCs, which may become a promising treatment in suppressing obesity-induced aging. Additionally, the above studies in humans signify that aging-related changes in hBMSCs might differ between overweight and obesity. This possibility should be explored by assessing the senescence of hBMSCs derived directly from overweight subjects in future studies.

## 5. Effect on Apoptosis of BMSCs

**Figure 1:**
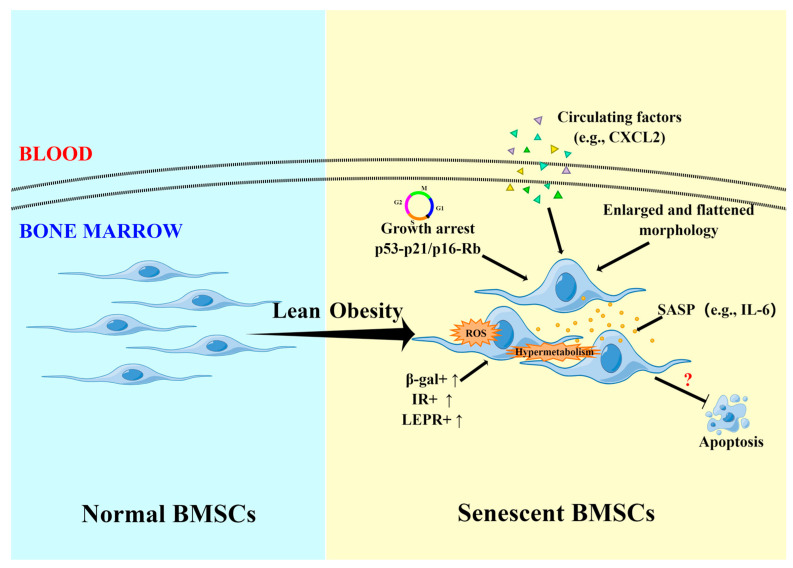
Obesity-associated senescent and apoptotic phenotype of BMSCs. Obese BMSCs are inclined to senescence, displaying enlarged and flattened morphology, cell cycle arrest, SASP, increased senescence-related markers, etc. However, obesity seems to have no significant effect on apoptosis of BMSCs. The exact underlying mechanism is still unknown. BMSCs: bone marrow-derived mesenchymal stem cells; CXCL2: C-X-C motif chemokine ligand 2; SASP: senescence-associated secretory phenotype; IL-6: interleukin 6; LEPR: leptin receptor; IR: insulin receptor; β-gal: senescence-associated β-galactosidase.

Apoptosis is a primary cellular mechanism to maintain tissue homeostasis [85]. Both insufficient and excessive apoptosis can lead to a series of diseases [86]. An animal experiment conducted by de Oliveira et al. [45] shows that HFD-induced obesity has a pro-apoptotic effect on Swiss mouse-derived bone marrow cells including BMSCs. Insulin-like growth factor-1 (IGF-1) treatment significantly improves the survival of obese BMSCs, and the apoptosis rate is close to normal. However, Alessio et al. [42] found no distinct difference in the apoptotic rate of BMSCs between the control and the obese mice. This result seems to suggest that obesity does not interfere with the apoptotic process of BMSCs. Similar results were also observed in the study on rats. Following the findings of Bi et al. [44], 4-month HFD-induced significantly increased body weight does not change the percentage of apoptotic BMSCs from female SD rats. Moreover, extending HFD to 6 months still has no effect on cell apoptosis. Interestingly, a study might even indirectly suggest that the apoptosis level of BMSCs in obese mice shows a trend to decrease [32]. In humans, insufficient studies focus on the apoptotic phenotype of overweight and obese hBMSCs. Only one study compares the effect of serum from overweight (BMI > 25 kg/m^2^) and healthy (BMI < 25 kg/m^2^) individuals on hBMSCs apoptosis, but no significant difference was detected [52]. Cellular senescence and apoptosis are two possible fates of BMSCs. Based on the current research, BMSCs under obesity conditions seem to be more inclined to senescence (obesity-related cellular senescence and apoptosis are shown in Figure 1). However, it is still unknown what mechanisms drive obesity-derived BMSC senescence instead of apoptosis.

## 6. Effect on Trilineage Differentiation Potential of BMSCs

The ability to differentiate into osteoblast, adipocyte, and chondrocyte in vitro is one of the most basic characteristics of BMSCs [87]. However, the trilineage differentiation potential of BMSCs under overweight and obesity conditions is also controversial.

### 6.1. Osteogenic Differentiation

Alessio et al. [42] demonstrated that BMSCs from obese and normal mice have similar mRNA levels of osteogenic markers (i.e., osteopontin (OPN), osterix (OSX)) when proliferating in vitro. In the absence of external differentiation cues, this finding might imply that obesity does not affect the osteoblast lineage commitment of BMSCs. Additionally, undisturbed in vitro osteogenic differentiation was also observed in obese BMSCs [42]. The same result can also be acquired from the study of Li et al. [50]. Based on alkaline phosphatase (ALP) expression and the level of mineralization in vitro, no significant change is induced by obesity in the osteogenic ability of BMSCs derived from normal and OVX female mice [50]. In contrast, several animal experiments show that obesity enhances the osteogenic potential of BMSCs. Compared with control mice, Cao et al. [31] found that obese mice have an increased number and total area of alkaline phosphatase-positive colonies (CFU-ALP+) and a higher level of ALP mRNA (no significant change in alpha 1 collagen (COL1A1) and osteocalcin (OCN) mRNA levels) in BMSCs after osteoinduction in vitro. These results are associated with the increased number and proliferation ability of osteogenic progenitors [31]. Moreover, obese BMSCs also show increased mineralization capacity, measured by the increased number and area of mineralized calcium nodules [31]. However, Shu et al. [35] reported that CFU-ALP+ colony number and mRNA levels of ALP in BMSCs do not show differences between obese and lean mice. Even so, obese BMSCs still possess higher transcription levels of runt-related transcription factor 2 (RUNX2), OSX and OCN, and enhanced ability to produce calcium nodules in vitro. In another study, the increased expression of *RUNX2*, the master regulatory gene of osteogenesis, is substantiated at the protein level in BMSCs derived from obese Wistar rats [34]. Lv et al. [33] also found that obese mice have elevated mRNA transcripts of RUNX2 in BMSCs (no significant change in OSX and distal-less homeobox 5 (DLX5) mRNA levels) compared with normal control mice. Moreover, the number of alizarin red-stained colonies formed by obese BMSCs significantly increases after in vitro osteogenic induction [33]. The results from the level of stem progenitor cells and molecules suggest that obesity increases the ability of BMSCs to differentiate into osteoblasts. Additionally, this study also explores the role of free fatty acids (FFAs)—a critical bridge between HFD and obesity—in the differentiation of BMSCs [88]. Direct treatment of FFAs (palmitic acid and oleic acid) on lean BMSCs has no significant effect on the expressions of osteogenesis-related genes. However, lean BMSCs exposed to conditioned medium from FFA-treated adipocytes exhibit increased expressions of osteogenic genes and elevated mineralization, which is consistent with the result of osteogenesis of obese BMSCs in vitro. These findings might reveal an underlying mechanism by which HFD-induced obesity promotes the osteogenic process of BMSCs. However, several other animal studies show that obesity inhibits the osteogenic potential of BMSCs [30,37,39,44,47,48,49]. Adhikary et al. [39] demonstrated that HFD-induced obesity severely impaired the mouse BMSCs’ mineralization ability with a 20% reduction of calcium nodules. In the study of Gautam et al. [37], obesity induced a decreased number of calcium nodules, even reaching approximately 50%. The ALP activity and mRNA level of COL1A1, RUNX2, and OCN in BMSCs of obese mice are suppressed as well [37]. However, the attenuated osteogenic ability can be improved by formononetin [37]. Recently, Benova et al. [48] and Chen et al. [49] also discovered that MSDC-0602K and asiatic acid can ameliorate the osteogenic ability of obese mouse-derived BMSCs, supported by increased mineralization, ALP activity, and osteogenic gene expression. Besides demonstrating diminished in vitro mineralization of BMSCs in obese mice, Wu et al. [30] further simulated an obese environment through adding high concentrations of palmitic acid, stearic acid, and oleic acid to the differentiation medium of lean BMSCs. However, the cells display significantly increased osteogenic ability, which does not fully reproduce the in vitro differentiation potential of obese BMSCs. Moreover, the phenotypic change under FFAs treatment is not the same as the result of lean BMSCs directly stimulated by FFAs in another experiment [33]. The different compositions of FFAs and research methods might be responsible for this discrepancy. By gene ontology pathway analysis, Ali et al. [47] found that obesity downregulates the expression level of osteoblast differentiation-related genes in BMSCs of OVX female mice. As for the reason for inhibited osteogenesis under obese conditions, Wang et al. [43] demonstrated that obesity can reduce the secretion of BMSC-derived exosomes and the level of carried LncRNA H19, thereby affecting the miR-467/HoxA10 axis and ultimately inhibiting the osteogenic process. Additionally, decreased chemerin in the bone marrow of obese mice is also associated with the suppressed osteogenesis of BMSCs [46].

In humans, McCann et al. [51] found that the percentage of the number and area of CFU-ALP+ formed by hBMSCs has no relationship with BMI, irrespective of females or males. These findings might imply that the proportion and proliferation of osteogenic progenitors are not affected by BMI. Tencerova et al. [29] demonstrated that obese hBMSCs have increased osteogenic ability in vitro, evidenced by increased ALP activity, matrix mineralization, and tissue-nonspecific alkaline phosphatase (ALPL) mRNA levels. Despite that, RNA sequencing and other experiments confirm that the molecular phenotype of obese hBMSCs shifts toward committed adipogenic progenitors. In contrast, Ulum et al. [28] found that high BMI (BMI > 30 kg/m^2^) reduces the mineralization capacity and ALPL transcript level of hBMSCs during in vitro osteoinduction. The abnormal osteogenesis is associated with elevated endoplasmic reticulum stress (ERS) and impaired unfolded protein response [28]. The administration of ERS inhibitors tauroursodeoxycholic acid (TUDCA) and 4-phenylbutyrate (4-PBA) can partially rescue osteogenic ability [28]. Compared with lean participants, Tencerova et al. did not find a significant change in mineralized nodule formation, ALP activity, and ALPL mRNA level of hBMSCs in overweight participants [29]. Nevertheless, after comparing hBMSCs treated by overweight (BMI > 25 kg/m^2^) and healthy (BMI < 25 kg/m^2^) serum, Di Bernardo et al. [52] believed that circulating signaling molecules from overweight serum partially damage osteogenic differentiation of BMSCs via changing the expression pattern of osteogenic genes (i.e., *OPN* and *OSX*), even though no significant difference was detected in the mineralized matrix.

### 6.2. Adipogenic Differentiation

Alessio et al. [42] demonstrated that the ability of obese BMSCs to differentiate into adipocytes in vitro is comparable with the cells from normal mice. Moreover, they also found that the expressions of adipogenic markers lipoprotein lipase and peroxisome proliferator-activated receptor γ (*PPARγ*) are not significantly changed in proliferating obese BMSCs, suggesting unaffected adipogenic lineage commitment under obesity status [42]. However, Cortez et al. [34] found that obesity downregulates the protein level of PPARγ in BMSCs of obese Wistar rats, which was considered to be associated with increased NF-κB expression. Likewise, the study by Lv et al. [33] also shows that obese mice have dramatically reduced mRNA and protein expressions of PPARγ in BMSCs compared with control mice. Moreover, the expression of preadipocyte factor-1 (PREF-1) mRNA is downregulated as well. Interestingly, the number of colonies stained by Oil Red O (ORO) in their differentiation experiments is not altered by obesity [33]. This discrepancy might indicate that inhibited adipogenic differentiation under obesity is from the molecular level instead of the stem progenitor cell level. Additionally, they also explored the direct and indirect effects of FFAs (palmitic acid and oleic acid) on the adipogenesis of BMSCs from mice [33]. Lean BMSCs directly treated with FFAs only exhibit decreased mRNA expression of PPARγ, yet the expressions of adipogenesis-related genes (*PPARγ*, CCAAT enhancer binding protein α (*C/EBPα*), and *PREF-1*) are suppressed in lean BMSCs when exposed to conditioned medium from FFA-treated adipocytes. Combined with the previously mentioned effects of FFAs on osteogenesis-related genes, obesity may regulate the differentiation of BMSCs through factors secreted by FFA-treated adipocytes [33]. In another study, Wu et al. [30] found that BMSCs from obese mice produce less fat in vitro than cells from lean mice. They also described the change in adipogenesis of lean BMSCs treated by increased FFAs (palmitic acid, stearic acid, and oleic acid). A high concentration of FFAs enhances the adipogenic ability of lean BMSCs, in disagreement with the findings of Lv et al. [30,33]. Culture methods might explain the inconsistent trend. In female SD rats, impaired adipogenesis of BMSCs induced by excess weight was also observed by Bi et al. [44], which is related to the upregulation of serum CXCL2. Further in vitro mechanism analysis shows that CXCL2 inhibits the adipogenic ability of BMSCs through Rac1 activation [44]. Nonetheless, some studies do not support the above conclusions and present opposite results. For instance, Adhikary et al. [39] demonstrated that obesity increases the fat production of mouse BMSCs after induction in vitro. At the molecular level, Tencerova et al. [38] found upregulated mRNA levels of adipogenic genes (e.g., PPARγ, leptin (LEP), adiponectin) in BMSCs of obese mice compared with BMSCs of lean mice, which indicates that obesity drives BMSCs toward adipogenic lineage commitment [38]. These phenotypes have been reproduced in several animal studies [35,37,48,49]. Furthermore, MSDC-0602K and asiatic acid contribute to the inhibition of enhanced adipogenesis induced by obesity [48,49].

In humans, Tencerova et al. [29] demonstrated that obesity enhances the in vitro adipogenesis of hBMSCs and shifts the fate of these cells towards adipocytic progenitors. These phenotypes associate with the enrichment of IR+ and LEPR+ cells which have high adipocyte differentiation capacity. In contrast, Ulum et al. [28] observed almost normal in vitro adipogenesis in BMSCs from donors with high BMI (BMI > 30 kg/m^2^), and no clear correlation exists between increasing BMI and adipogenic potential. Yet the change in mRNA levels of adipogenic genes (e.g., PPARγ, LEP) during differentiation proves a relative defect of high-BMI hBMSCs in adipogenesis [28]. TUDCA and 4-PBA are found to facilitate adipogenic differentiation [28]. Compared with hBMSCs of lean donors, Tencerova et al. [29] proposed that hBMSCs of overweight donors have similar in vitro adipogenic ability. However, Di Bernardo et al. [52] showed that overweight serum (BMI > 25 kg/m^2^) enhances the adipogenic potential of BMSCs as evidenced by increased mature adipocytes and upregulated adipogenesis-related markers (e.g., *PPARγ*, *C/EBPα*).

### 6.3. Chondrogenic Differentiation

SRY-box transcription factor 9 (SOX9) is the critical regulator committing BMSCs to the chondrogenic lineage. Compared with low diet-fed mice, Shu et al. [35] demonstrated that the SOX9 mRNA level of BMSCs increases (no significant change in type II collagen mRNA level) in HFD-induced obese mice. However, Lv et al. [33] found that the mRNA level of SOX9 in BMSCs is not significantly different between normal control and obese mice. Interestingly, the expression of SOX9 mRNA in BMSCs of lean mice is also not changed after being directly treated by FFAs (palmitic acid and oleic acid), while conditioned medium from FFA-treated adipocytes upregulates *SOX9* expression in lean BMSCs [33]. In another study, Alessio et al. [42] did not find significant effects of obesity on the protein levels of chondrogenic markers (e.g., aggrecan) and in vitro chondrogenesis of BMSCs in mice. By comparison, Wu et al. [30] proposed that obesity inhibits the chondrogenic ability of mouse BMSCs in vitro, exhibiting a reduced glycosaminoglycan (GAG)/DNA ratio and production of GAGs and type II collagen. The findings might imply that obesity limits the application of BMSCs in cartilage repair. Additionally, Wu et al. [30] also confirmed no impact of FFAs (palmitic acid, stearic acid, and oleic acid) on the chondrogenic property in vitro of BMSCs harvested from lean mice. In contrast to animal study, there are no data for chondrogenesis of hBMSCs under overweight/obese status, which are required in the future.

## 7. Conclusions

In this review, the effects of overweight and obesity on the proliferation, clonogenicity, surface antigen expression, senescence, apoptosis, and trilineage differentiation of BMSCs were summarized. Currently, more evidence originates from HFD-induced obese animal models. The results, however, are inconsistent partly due to different methodological approaches. Likewise, different reports also exist in studies on human BMSCs. The reasons may come from overweight or obesity itself and interference from other comorbidities. Moreover, animal models cannot fully capture the complex pathophysiological environment of the human body. Therefore, it is difficult to identify the exact mechanisms underlying the phenotypes. Although the positive impacts of overweight and obesity on BMSCs in some respects cannot be completely denied, their side effects are still an inevitable huge challenge for future clinical applications. Thus, rigorous evaluation is needed for BMSCs from overweight and obese donors to minimize the impact of unexpected consequences, and allogeneic BMSC therapies might be better for overweight and obese patients. In addition, there is insufficient research to explore how to reverse the unhealthy state of BMSCs caused by overweight and obesity. In other words, it is urgent to elucidate whether weight loss or ameliorating obesity-related metabolic disorders can improve the functions of BMSCs. Therefore, more high-quality research and evidence are required to further understand the influence of overweight and obesity on the properties of BMSCs, optimize BMSC products, and develop new treatment strategies.

## Figures and Tables

**Table 1:** Animal studies included in the review.

Author	Species	Gender	Age	Diet/Duration	BMSC Origin	Main Conclusion(s)
Cao et al. [31]	C57BL/6 mice	Male	6 weeks + 3 days	A control purified diet (10% energy as fat, D12450B, Research Diet, Inc., New Brunswick, NJ, United States) based on AIN-93G or an HFD (45% energy as fat) with extra fat from lard for 14 weeks	Tibias and femurs	Increased osteogenic potential of obese BMSCs
Lv et al. [33]	C57BL/6 mice	Male	2 weeks	A normal chow or an HFD for 24 weeks	Tibias and femurs	Enhanced osteogenesis and inhibited adipogenesis in obese BMSCs, which might be associated with factors secreted by FFA-treated adipocytes
Cortez et al. [34]	Wistar rats	Male	2 months + 10 days	A control diet (AIN-93M diet; total energy: 75.8% carbohydrates, 9.3% fat, and 14.9% protein) or an HFD (an AIN-93M-based diet that was enriched with lard; total energy: 24.2% carbohydrates, 60.9% fat, and 14.9% protein) for 12 weeks	Femurs	Increased inflammatory cytokine (i.e., IL-1, IL-6, and TNF-α) secretion, upregulated NF-κB and RUNX2 expression, and reduced PPARγ expression in obese BMSCs
Wu et al. [30]	C57BL/6J mice	Male	—	A low-fat diet (D12450B, 10% energy from fat, Research Diets, Inc.) or an HFD (D12492, 60% energy from fat, Research Diets, Inc.) for 14 weeks	Tibias and femurs	No significant change in proliferation; reduced trilineage differentiation, decreased CD105, and increased PDGFRα expression in obese BMSCs; FFAs increase adipogenic and osteogenic differentiation and have no impact on chondrogenic differentiation of BMSCs
Shu et al. [35]	C57BL/6J mice	Male	5 weeks	A low-fat diet (10% kcal, D12450B, Research Diets, Inc.) or an HFD (60%kcal, D12492, Research Diets, Inc.) for 12 weeks	Tibias and femurs	No significant difference in clonogenicity between obese and lean BMSCs; increased adipogenic and osteogenic differentiation abilities of obese BMSCs; increased SOX9 mRNA level in obese BMSCs
da Silva et al. [36]	C57BL/6 mice	Male	4 weeks	A regular chow (396 kcal/100g, 13% of energy derived from fat) or an HFD (470 kcal/100 g, 45% of energy derived from fat) for 10 weeks	Tibias and femurs	Obesity increases the proliferation of BMSCs and commits these cells to adipogenesis; similar expressions of BMSC markers between lean and obese mice
Gautam et al. [37]	C57BL/6 mice	Male	4 weeks	A standard chow diet or an HFD (D12109, 40% kcal% fat; Research Diets, Inc.) or an HFD (D12109, 40% kcal% fat; Research Diets, Inc.) and formononetin (0.1,1,10 mg/kg per day) for 12 weeks	Femurs	Formononetin improves osteogenic differentiation and inhibits adipogenic differentiation of obese BMSCs
Picke et al. [32]	C57BL/6J mice	Male	4–5 weeks	An HFD (EF R/M D12331 diet modified by Surwit, ssniff) or a chow diet for 12 to 20 weeks Note: CD90 knock-out mice on C57BL/6J background and wild-type C57BL/6J mice for functional validation	Tibias and femurs	The similar clonogenic ability between lean and obese BMSCs; reduced CD90 expression in obese BMSCs; CD90-deficient BMSCs have increased adipogenic differentiation, attenuated osteogenic differentiation, and a decreasing trend in apoptosis
Tencerova et al. [38]	C57BL/6J mice	Male	8 weeks	A normal chow diet (Altromin, Lage, Germany, 132003, containing 6% fat, 30% protein, 63% carbohydrate, 7.7% sucrose) or a 60 kcal% high-fat diet (Research Diet, Inc., D12492, containing 35% fat, 26% protein, 26% carbohydrate, 8.8% sucrose) for 12 weeks	The bones of the front and hind limbs	Inhibited proliferation and colony-forming potential in obese BMSCs; downregulated expressions of CD73+ and Sca1/CD140a+ and upregulated adipogenesis-associated genes in obese BMSCs; no significant change in senescent phenotype
Adhikary et al. [39]	BALB/c mice	Male	9 weeks	A standard chow diet or an HFD (D12492, 60% energy from fat; Research Diets, Inc.) for 10 weeks	Femurs	Impaired mineralization ability and increased adipogenic differentiation in obese BMSCs
Ayaz-Guner et al. [40]	C57BL/6 mice	Male	3 weeks	A normal diet (containing 10% fat, 70% carbohydrates, and 20% protein (total 3.82 kcal/g)) or an HFD (containing 60% fat from lard, 20% carbohydrates, and 20% protein (total 5.21 kcal/g), Research Diets, Inc.) for 10 weeks	Tibias and femurs	Comparable expressions of CD105, CD90, and CD73 between normal and obese BMSCs
Li et al. [41]	Wild-type and IL-6-deficient mice generated on C57BL/6 background	Male	8 weeks	A standard diet (19.2% protein, 67.3% carbohydrate, 4.3% fat) or an HFD (26% protein, 26% carbohydrate, 35% fat) for 12 weeks	Tibias and femurs	Obesity attenuates the clonogenic ability and induces senescence of BMSCs, which can be ameliorated by *IL-6* knockout
Alessio et al. [42]	C57BL/6 mice	Male	3 weeks	A normal diet (10% fat, 70% carbohydrate, and 20% protein (total 3.82 kcal/g)) or an HFD (containing 60% fat from lard, 20% carbohydrate, and 20% protein (total 5.21 kcal/g), Research Diets, Inc.) for 10 weeks	Tibias and femurs	Suppressed proliferation, increased senescence, unchanged apoptosis, clonogenicity and trilineage differentiation in obese BMSCs
Wang et al. [43]	C57BL/6J mice	Male	7 weeks	A normal diet or an HFD	Tibias and femurs	Obesity reduces the secretion of BMSC-derived exosomes and the level of carried LncRNA H19, thereby affecting the miR-467/HoxA10 axis and ultimately inhibiting the osteogenic process
Bi et al. [44]	Sprague-Dawley rats	Female	6 weeks	A normal diet (GB14924.3-2001, 100% basal diet) and a customized HFD (containing 60% basal diet, 10% sucrose, 20% lard, and 10% egg yolk powder) for 4 months and 6 months	Tibias and femurs	Inhibited proliferation, unchanged apoptosis, reduced adipogenic and osteogenic differentiation, increased senescence in obese BMSCs; CXCL2 impairs adipogenesis and promotes senescence of BMSCs
de Oliveira et al. [45]	Swiss mice	Male	21 days	A standard AIN93G diet (65.6% carbohydrates, 17.3% proteins, and 17.1% lipids) or a Western diet rich in saturated fat and simple carbohydrate from clarified butter (Ghee) (43.3% carbohydrates, 14% proteins, and 42.7% lipids) (PragSoluções, Brazil) for 12 weeks Note: After 11 weeks, half of the obese mice received IGF-1 treatment.	Tibias and femurs	Obesity has a pro-apoptotic effect on BMSCs, which can be improved by IGF-1
Li et al. [46]	C57BL/6 mice	Male	8 weeks	A normal chow or an HFD (60% kcal in fat) for 12 weeks Note: Chemerin knock-out (Rarres2−/−) and Ap2−drive Rarres2 overexpression transgenic mice for functional validation	Tibias and femurs	Decreased chemerin in the obese bone marrow is associated with the suppressed osteogenesis of BMSCs
Ali et al. [47]	C57BL/6J mice and OVX C57BL/6J mice	Female	8 weeks	A R-70 normal diet (Lantmännen, containing kcal%: protein 14.5%, carbohydrates 60%, and fat 4.5%) in which fat content was from oatmeal, barley, wheat bran, wheat flour or an HFD (Research Diet, D12492 containing kcal%: protein 20%, carbohydrates 20%, and fat 60%) in which fat content was from soybean oil and lard for 12 weeks	The bones of the front and hind limbs	Obesity upregulates senescence-related genes and downregulates osteogenic genes in BMSCs of OVX mice
Benova et al. [48]	C57BL/6N mice	Male	12 weeks	A normal diet (3.4% wt/wt as lipids) or an HFD (lipid content, ~35% wt/wt, mainly corn oil) or an HFD and pioglitazone (50 mg pioglitazone/kg diet (Actos, Takeda, Japan)) or a HFD+MSDC-0602K (330 mg MSDC-0602K/kg diet (Cirius Therapeutics, USA)) for 8 weeks	The bones of the front and hind limbs	No significant difference in proliferation and colony-forming ability between lean and obese BMSCs; MSDC-0602K increases osteogenesis and reduces adipogenesis of obese BMSCs
Chen et al. [49]	C57BL/6 mice	Male	4 weeks	A normal control diet or an HFD (carbohydrates, 20.3%; fat, 61.6%; protein, 18.1%) or an HFD with 25 mg/kg asiatic acid or an HFD with 50 mg/kg asiatic acid.	Femurs	Asiatic acid ameliorates inhibited osteogenesis and suppresses enhanced adipogenesis of obese BMSCs
Li et al. [50]	C57BL/6 mice and OVX C57BL/6 mice	Female	3 months	A standard diet or an HFD for 8 weeks	Tibias and femurs	Obesity has no significant effect on the osteogenic ability of BMSCs from normal and OVX female mice

Notes: BMSCs: bone marrow-derived mesenchymal stem cells; HFD: high-fat diet; FFA: free fatty acid; IL-1: interleukin 1; IL-6: interleukin 6; TNF-α: tumor necrosis factor-α; RUNX2: runt-related transcription factor 2; PPARγ: lipoprotein lipase and peroxisome proliferator-activated receptor γ; PDGFRα: platelet-derived growth factor receptor α; SOX9: SRY-box transcription factor 9; IGF-1: Insulin-like growth factor-1; OVX: ovariectomized.

**Table 2:** Human studies included in the review.

Author	Gender	Age	BMI	BMSC Origin	Main Conclusion(s)
McCann et al. [51]	Male and Female	28–91 years (mean age 64 years and median age 63.5 years)	Male: mean BMI: 29.6 kg/m^2^ median BMI: 29.3 kg/m^2^ Female: mean BMI: 28.1 kg/m^2^ median BMI: 27.4 kg/m^2^	Femoral canal	BMI is positively correlated with colony area and number in males but not females; the percentage of the number and area of CFU-ALP+ has no relationship with BMI, irrespective of females or males
Di Bernardo et al. [52]	Male	BMSCs from 10-year-old, 12-year-old, and 13-year-old male donors; Sera from healthy and overweight adult males	Healthy weight: 21.10 ± 1.10 kg/m^2^ Overweight: 29.63 ± 1.80 kg/m^2^	—	Overweight (BMI > 25 kg/m^2^) serum does not influence the proliferation, senescence, and apoptosis of hBMSCs; overweight (BMI > 25 kg/m^2^) serum increases adipogenesis and impairs osteogenesis of hBMSCs
Ulum et al. [28]	—	Normal BMI: 8–36 years High BMI: 14–58 years	Normal BMI: 17.62 ± 4.27 kg/m^2^ High BMI: 30.85 ± 4.16 kg/m^2^	—	High-BMI (BMI > 30 kg/m^2^) hBMSCs have several altered expressions of surface antigens, decreased proliferation, increased senescence, impaired osteogenesis, and a relative defect in adipogenesis; the abnormal osteogenesis is associated with elevated ERS and impaired unfolded protein response; TUDCA and 4-PBA can partially rescue the osteogenic ability and facilitate adipogenesis of high-BMI (BMI > 30 kg/m^2^) hBMSCs
Tencerova et al. [29]	Male	Lean: 31 ± 3 years Overweight: 32 ± 3 years Obese: 37 ± 2 years	Lean: 22.9 ± 0.3 kg/m^2^ Overweight: 28.0 ± 0.4 kg/m^2^ Obese: 36.1 ± 0.8 kg/m^2^	Iliac crest	Obese hBMSCs inhibited proliferation and clonogenicity; enhanced in vitro adipogenic and osteogenic differentiation; increased number of LEPR+, IR+, and senescent cells; increased commitment to adipogenesis Overweight hBMSCs decreased proliferation; unchanged clonogenicity, in vitro adipogenic and osteogenic differentiation

Notes: BMI: body mass index; BMSCs: bone marrow-derived mesenchymal stem cells; hBMSCs: human bone marrow-derived mesenchymal stem cells; ERS: endoplasmic reticulum stress; TUDCA: tauroursodeoxycholic acid; 4-PBA: 4-phenylbutyrate; LEPR: leptin receptor; IR: insulin receptor.

## Data Availability

Not applicable.
